# Generation and Evaluation of an IPTG-Regulated Version of *Vav*-Gene Promoter for Mouse Transgenesis

**DOI:** 10.1371/journal.pone.0018051

**Published:** 2011-03-21

**Authors:** Francesca Grespi, Eleonora Ottina, Nikolaos Yannoutsos, Stephan Geley, Andreas Villunger

**Affiliations:** 1 Division of Developmental Immunology, Medical University Innsbruck, Innsbruck, Austria; 2 Division of Cell Biology, Medical University Innsbruck, Innsbruck, Austria; 3 Division of Molecular Pathophysiology, Innsbruck Biocenter, Medical University Innsbruck, Innsbruck, Austria; Brigham and Women's Hospital, United States of America

## Abstract

Different bacteria-derived systems for regulatable gene expression have been developed for the use in mammalian cells and some were also successfully adopted for *in vivo* use in vertebrate model organisms. However, certain limitations apply to most of these systems, including leakiness of transgene expression, inefficient transgene silencing or activation, as well as limited tissue accessibility of transgene-inducers or their unfavourable pharmacokinetics. In this study, we evaluated the suitability of the *lac*-operon/*lac*-repressor (*lacO/lacI*) system for the regulation of the well-established *Vav*-gene promoter that allows inducible transgene expression in different haematopoietic lineages in mice. Using the fluorescence marker protein Venus as a reporter, we observed that the *lacO/lacI* system could be amended to modulate transgene-expression in haematopoietic cells. However, reporter expression was not uniform and the *lacO* elements introduced into the *Vav*-gene promoter only conferred limited repression and reversion of *lacI*-mediated gene silencing after administration of IPTG. Although further optimization of the system is required, the *lacO*-modified version of the *Vav*-gene promoter may be adopted as a tool where low basal gene-expression and limited transient induction of protein expression are desired, *e.g.* for the activation of oncogenes or transgenes that act in a dominant-negative manner.

## Introduction

Transgenesis in mice has become a useful tool to study gene function and model human diseases *in vivo*. Examples of transgenic mouse strains generated to study oncogenesis in the haematopoietic system include, amongst others, mice overexpressing anti-apoptotic *Bcl-2* to model follicular B cell lymphomas, a mutated version of *N-Ras* driving T cell and histocytic lymphomagenesis, the *BCR-abl* fusion protein driving chronic myelocytic leukemia and cases of acute myelocytic leukemia in humans or the *c-Myc* proto-oncogene under the control of the Ig-heavy chain enhancer (*Eµ*) that develop aggressive preB and IgM^+^ B cell lymphomas, mimicking to a certain degree features of Burkitt lymphoma [1].

Although transgenic mice are suitable models to study a variety of pathological states, certain restrictions apply. One of the problems connected with transgene overexpression is putative cytotoxicity, sometimes associated with induced lethality, but more frequently silencing of transgene expression and counter-selection of cells with low or no transgene expression. Another limitation is related to the fact that expression of the target gene may be only desired in a specific cell type, at a specific developmental stage or for a limited time frame to better mimic events during normal development or human disease pathology. To overcome these problems, tissue specific transgenesis has been developed that aims to exploit certain regulated gene-expression systems derived from bacteria, e.g. the tetracycline-based TetON/OFF system developed by Bujard and colleagues [2], or, for nuclear acting transgenes such as *Cre* recombinase, estrogen-receptor (ER)-fusion proteins that can be retained in the cytoplasm and translocate into the nucleus upon application of the synthetic ligand, 4-hydroxytamoxifen (4-OHT) [3]. Although well established in cell lines and today frequently used in transgenic mouse strains, certain limitations apply to these systems, mainly insufficient tightness of gene-repression and/or moderate induction levels, e.g., due to ineffective delivery and targeting of agonists to the cell type/tissue of interest, as well as stochastic epigenetic transgene silencing [4]–[5].

Therefore, we aimed to combine a tissue-specific transgene expression system with an inducible one that would allow regulated transgenesis in the haematopoietic system. We chose the promoter of the *Vav* gene, expressed in the entire hematopoietic lineage but few other cell types, showing good expression levels in all cell types of the blood, including multipotent progenitors as well as haematopoietic stem cells [6]. This promoter has been already used successfully for the expression of *Bcl-2* [7], *Mcl-1* [8] or *Cre*-recombinase [9] in the hematopoietic compartment and is most suitable when consequences of transgene expression need to be studied in the context of more than just a single haematopoietic cell type. To generate a system that may also provide tight and reversible control of the *Vav*-gene promoter we chose to explore the suitability of the *lac* repressor/operator (*lacI/lacO*) system previously be shown to allow timed and reversible transgene expression in mice [10], [11]. Insertion of three *lacO* sites into the *VavP* transgenic vector did allow expression of a fluorescent reporter protein, Venus, in a manner comparable to unmodified *VavP* promoter. Notably, reporter expression appeared variegated/mosaic in different haematopoietic cell types but was strongly reduced in double-transgenic mice in which the *lac* repressor was expressed ubiquitously under the control of the human *β-actin* promoter [10]. However, the efficiency of re-expression of the reporter in different cell types was highly variable and cell type dependent, indicating and the need for further optimization for satisfying use in haematopoietic cells *in vivo*.

## Results

### 1) Insertion of lacO elements into the *VavP* vector confers IPTG sensitivity

**Figure 1:**
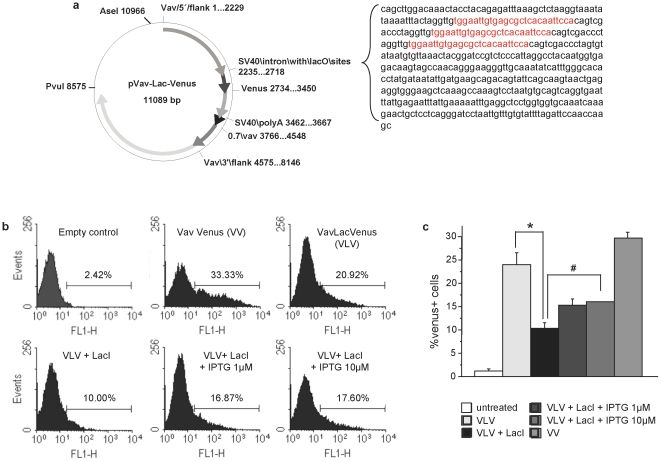
Generation of an IPTG-responsive version of the *Vav*-transgenic vector. (**a**) Schematic representation of the modified *VavP* HS21/45 plasmid (VLV) where three lac repressor binding sites (lacO, sequence in red) were introduced in the SV40 intronic sequence upstream of the multiple cloning site, bearing the *Venus* cDNA. (**b**) Functionality of the construct was assessed before oocyte-injection by transient transfection into 293 T cells in the presence or absence of a construct encoding for the *lacI* repressor followed by flow cytometric analysis. (**c**) 293 T cells transfected with empty vector, VV, VLV ± lacI were exposed to different doses of isopropyl β-D-1-thiogalactopyranoside (IPTG) and 24 h later the percentage of Venus^+^ cells was quantified by flow cytometric analysis. Bars represent means ± SEM of a representative experiment performed in triplicate *p<0,01, #p<0.05.

We chose to adopt the *Vav*-gene promoter, demonstrated to confer tissue specific expression of transgenes in vivo [6], in order to make it amenable to timed regulation. A DNA element derived from the pOPI3CAT expression vector containing three symmetric *lac* operator (*lacO*) binding sites recognized by the *lac* repressor (*lacI*), within a SV40-derived intron, was subcloned between the transcription start site of the endogenous *Vav*-gene and the multiple cloning site (MCS) of the HS21/45 *VavP*-transgenic vector. Venus, an optimized version of GFP [12], was inserted as a reporter into the MCS (Fig. 1a). The insertion was confirmed by sequencing and the *VavlacOVenus* (VLV) construct was tested for functionality by transient transfection into 293T cells, either alone or together with a plasmid encoding the codon optimized mammalian version of the *lacI* [10]. Inspection of cells in an immunofluorescence microscope followed by flow cytometric analysis confirmed expression of Venus in a subset of cells transfected with the VLV plasmid alone that was reduced when co-expressed along with a *lacI*-encoding plasmid (Figs. 1b,c). Quantification by flow cytometry revealed that co-expression of *lacI* indeed reduced the percentage of Venus^+^ cells from ∼24% to ∼10%, while addition of the synthetic inducer IPTG lead to a significant (p<0.05) increase in Venus expression (Fig. 1c). Increasing the concentration of IPTG further to 0.1 or 1 mM did not significantly improve the efficacy of re-induction, consistent with published results using embryonic R3 cells [10]. Venus encoding cDNA was also subcloned into an unmodified version of the *Vav*-transgenic vector (VV) that served as a control. The percentage of Venus expressing 293T cells was comparable between VLV and VV transfected cells (Fig. 1c) demonstrating that the insertion of the SV40-intron did not compromise the activity of the *Vav* promoter, at least *in vitro*.

### 2) Generation of *VavLacOVenus* transgenic mice

**Figure 2:**
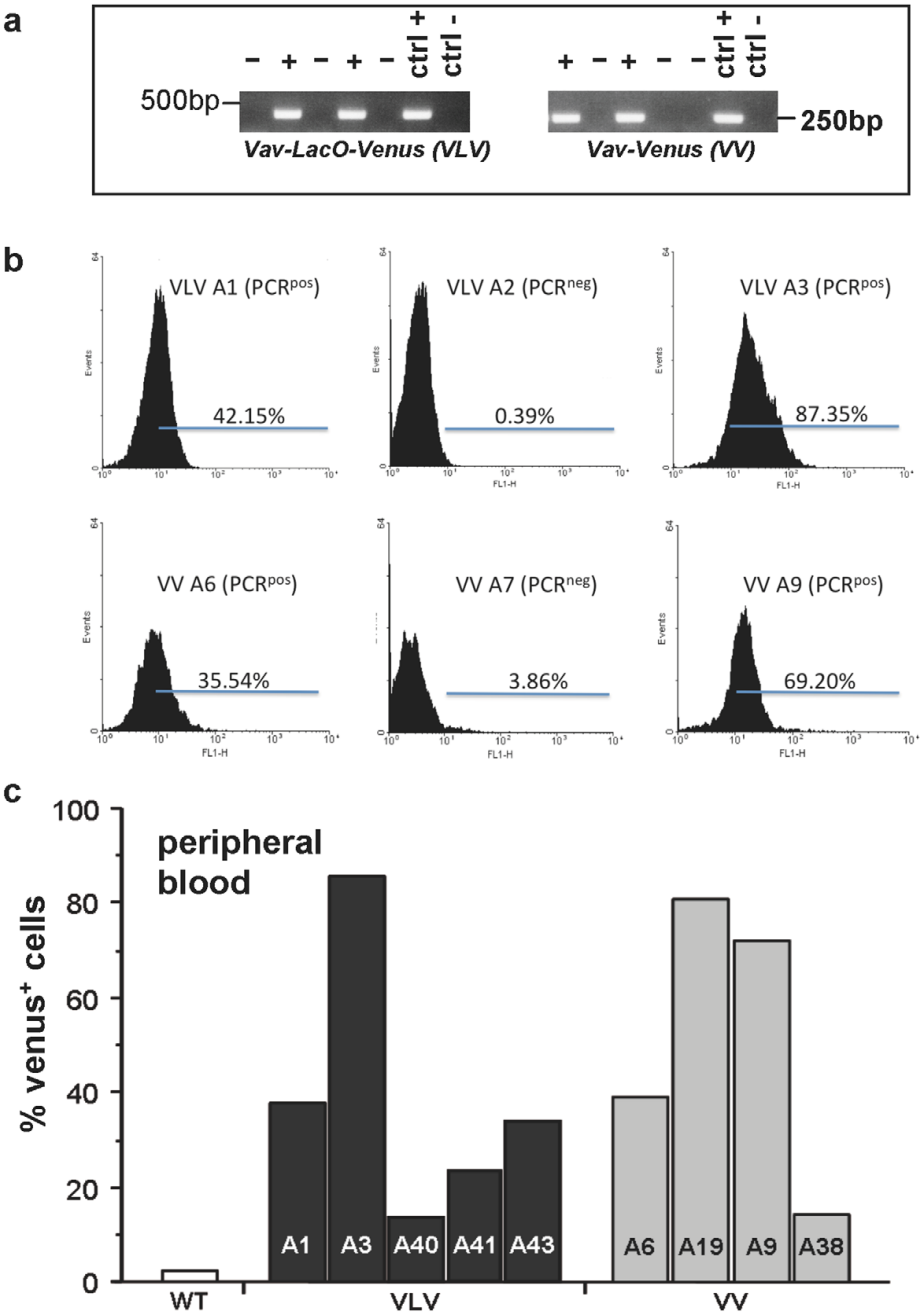
Identification of transgenic mice. The *VavLacOVenus* (VLV) plasmid as well as an unmodified version of the *VavP* plasmid encoding *Venus* cDNA (VV) were digested with AseI and PvuI and an approximately 9.2 kb AseI-PvuI fragment was used for microinjection into fertilized FVB oocytes. (**a**) Transgenic founders were first identified by PCR genotyping on tail DNA. VLV  =  *VavLacOVenus* transgenic mouse, VV  =  *VavVenus* transgenic mouse, WT  =  wild type FVB mouse. (**b**) Histograms from flow cytometric analysis showing different levels of transgene expression in PCR-typed founders. (**c**) Range of transgene expression levels in PCR^+^ founders, quantified as in (b).

Transgenic mice were generated by microinjection of the VLV or VV constructs isolated as a linear fragment into fertilized oocytes from FVB mice. PCR genotyping on tail DNA using primers specific for *Venus* cDNA and *VavP* cassette sequence was performed to identify founders carrying the transgene (Fig. 2a). Since transgene insertion does not necessarily result in its expression, e.g. due to silencing or positioning effects in heterochromatin, we collected peripheral blood (PB) from PCR^+^ and PCR^−^ mice and performed flow cytometric analysis to verify the typing results and quantify the percentage of Venus-expressing cells. Venus expression in the peripheral blood varied significantly between founders, indicating that some of the founders were mosaic or may show variegated transgene expression e.g., due to positioning effects of the transgene (Fig. 2b,c). Out of the transgene-expressing founders, we chose those showing highest Venus expression for further breeding, i.e. VLV A1 and A3 as well as VV A9 and A19. All experiments shown below are derived using VLV A3 and VV A9 derived progeny, respectively. Similar results regarding heterogeneous transgene expression were also observed in the offspring of other founders (not shown).

### 3) Expression of Venus protein is detectable in myeloid and lymphoid cells but appears variegated

**Figure 3:**
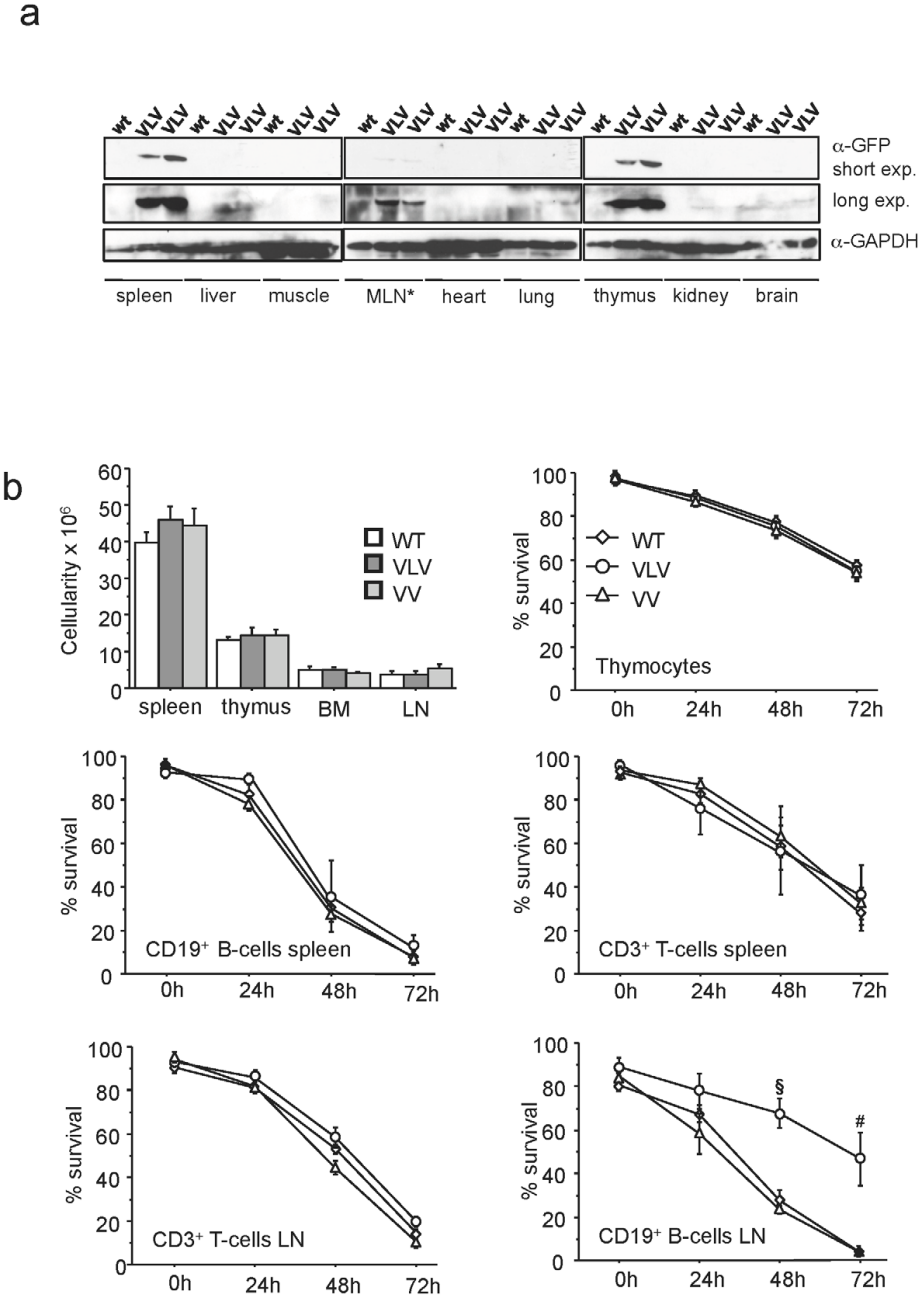
Characterization of Venus expression, organ cellularity and lymphocyte survival *in vitro*. (**a**) Protein lysates (55 µg/lane) from the indicated organs harvested from wt or VLV mice were separated by SDS-PAGE, transferred onto nitrocellulose membranes and immunoblotting was performed using an antiserum generated against GFP. Membranes were reprobed using a monoclonal antibody recognizing GAPDH to demonstrate comparable protein loading. *MLN  =  mesenteric lymph nodes. (**b**) Mice of the indicated genotpyes 6-8 weeks of age were sacrificed to evaluate organ cellularity (n = 4/genotype). Primary cells derived from total spleen, thymus or lymph nodes were put in culture and spontaneous apoptosis was quantified by flow-cytometry. Markers for T- (CD3) and B-cells (CD19) were used in combination with AnnexinV-APC+7-AAD staining to monitor survival over time. Data points represent means ± SEM of three independent experiments (§  =  p>0.001 VLV vs VV and WT, #  =  p>0.01 VLV vs VV and WT).

Although the transgenic animals did not show any overt phenotype up to an observation period of 6 month, we wanted to monitor whether overexpression of Venus could have some impact on lymphocyte number or survival, since high level expression of GFP has been reported to cause some toxicity in cultured cells [13] and reportedly correlated with premature lethality when overexpressed strongly in cardiomyocytes [14]. First, we performed Western blot analysis on different tissues that confirmed restriction of transgene expression to haematopoietic organs (Fig. 3a). Next, we quantified leukocyte numbers in haematopoietic organs and compared transgenic lines with littermate controls that failed to reveal any significant differences (p>0.2) in cell number (Fig. 3b). Second, we put primary lymphocytes derived from thymus, spleen or lymph nodes in culture and monitored cell survival by Annexin V/PI staining, in combination with cell surface marker staining to identify T and B cells, over time. Thymocytes as well as mature B- and T-cells derived from the spleen of the VLV or VV mice did not show any difference in survival in culture when compared to those ones derived from wt mice (Fig. 3b). Unexpectedly, the B-cells derived from the lymph nodes of VLV mice appeared more resistant to spontaneous apoptosis than the ones of VV or wt mice that died with similar kinetics (Fig. 3b). Together our results show that Venus expression is well tolerated in lymphocytes over time *in vivo*. Also, in the VLV strain chosen for detailed analysis, transgene insertion may influence expression/function of gene(s) associated with the survival of mature B cell, at least *in vitro*. However, since we did not observe B cell accumulation *in vivo* this observation was not followed up in detail.

**Figure 4:**
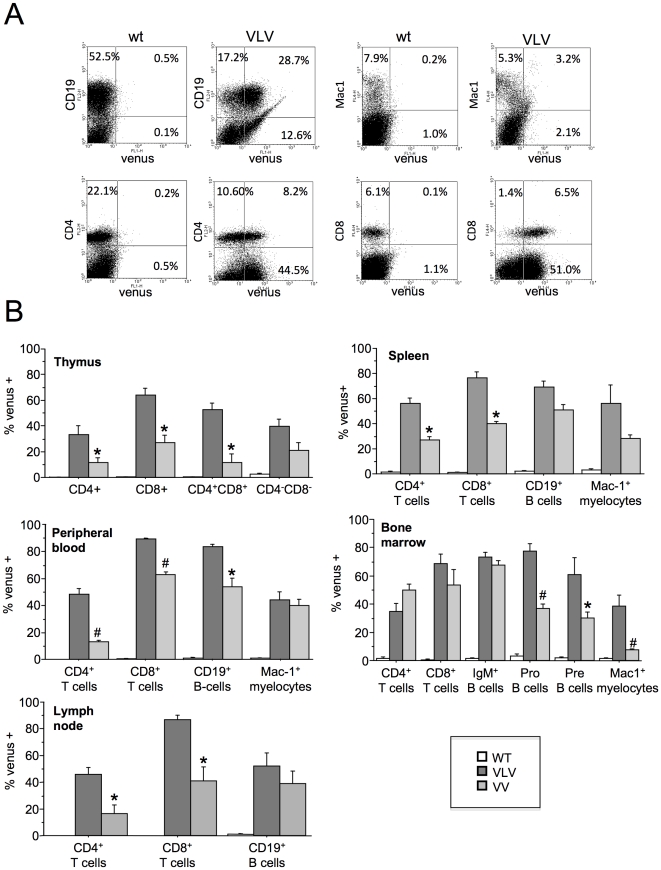
Characterization of transgene expression in haematopoietic cells from VLV and VV mice. Animals that proved positive for Venus expression in the peripheral blood were sacrificed for further analysis of transgene expression in different organs. (**a**) Single cell suspension from spleens were stained with fluorochrome labeled antibodies specific for cell surface markers identifying T cells (CD4, CD8), B cells (CD19), as well as myelocytes (Mac-1) and subjected to flow cytometric analysis. Representative dot blots showing variegate expression of the transgene are shown. (**b**) Summary of the experiments shown in (a), performed to assess the percentage of transgene expressing leukocytes in all major haematopoietic organs. Bars represent means ± SEM (wt n = 8, VLV n = 5, VV = 3); *p<0.001; #p<0.05. P values refer to significant differences in Venus expression between cells derived from VLV and VV mice. F2 generation offspring of VLV founder A3 and F3 generation offspring of VV founder A9 were used for this analysis, as well as transgene-negative littermate controls of both strains.

We continued our analysis quantifying the percentage of Venus^+^ cells in different primary and secondary lymphatic organs. Therefore, we stained single cell suspensions with antibodies specific for different cell surface markers, identifying T-cells, B-cells or myelocytes and performed flow cytometric analysis. Venus^+^ cells were found in all the leukocyte subpopulations tested. However, the relative percentage of Venus^+^ cells varied between the individual transgenic lines as well as between littermates, indicating variegated expression of the reporter or mosaicism due to stochastic gene silencing. Similar observations were made in all other lymphoid organs analyzed (Fig. 4a). In VLV transgenic mice, T cells showed Venus expression in all the organs ranging from 35%–80%, with highest expression found in CD8^+^ T cells in lymph nodes and spleen (∼80%), while the percentage of Venus^+^ CD4^+^ T cells was frequently lower in thymus, peripheral blood, spleen, lymph node and bone marrow (p<0.03) (Fig. 4b). In the CD19^+^ B cell compartment in the periphery, we found that transgene expression was actually highly comparable between spleen, peripheral blood and bone marrow with 70–80% of Venus expressing B cells, but only about half of the B cells in the lymph node were expressing the transgene (Fig. 4b). Immature pro- and pre-B-cells in the bone marrow also expressed Venus, with a slightly higher percentage of transgene positive pro-B than pre-B cells (Fig. 4b). The percentage of Venus^+^ Mac1^+^ myelocytes was comparable to the percentage of Venus^+^ lymphocytes in the spleen, while it was significantly lower in the bone marrow and peripheral blood (p<0.002) of VLV mice (Fig. 4b). Comparing levels of Venus^+^ cells in the VLV with those in VV transgenic mice we noticed an overall similar pattern of transgene expression but a generally lower percentage of Venus^+^ cells in the VV strain (Fig. 4b). This phenomenon is most likely due to different sites of insertion of the transgene and/or copy number variation. Notably, qPCR analysis performed on tail DNA derived from three randomly picked animals of each strain revealed an about 2.5-fold higher signal for Venus in the VV samples, indicating higher copy number in this strain (not shown). This suggests that chromatin effects at the site of integration rather than copy number accounts for the difference in transgene expression between the two strains.

### 4) Gene-silencing of Venus expression in *VavLacOVenus/LacI* double transgenic mice

**Figure 5:**
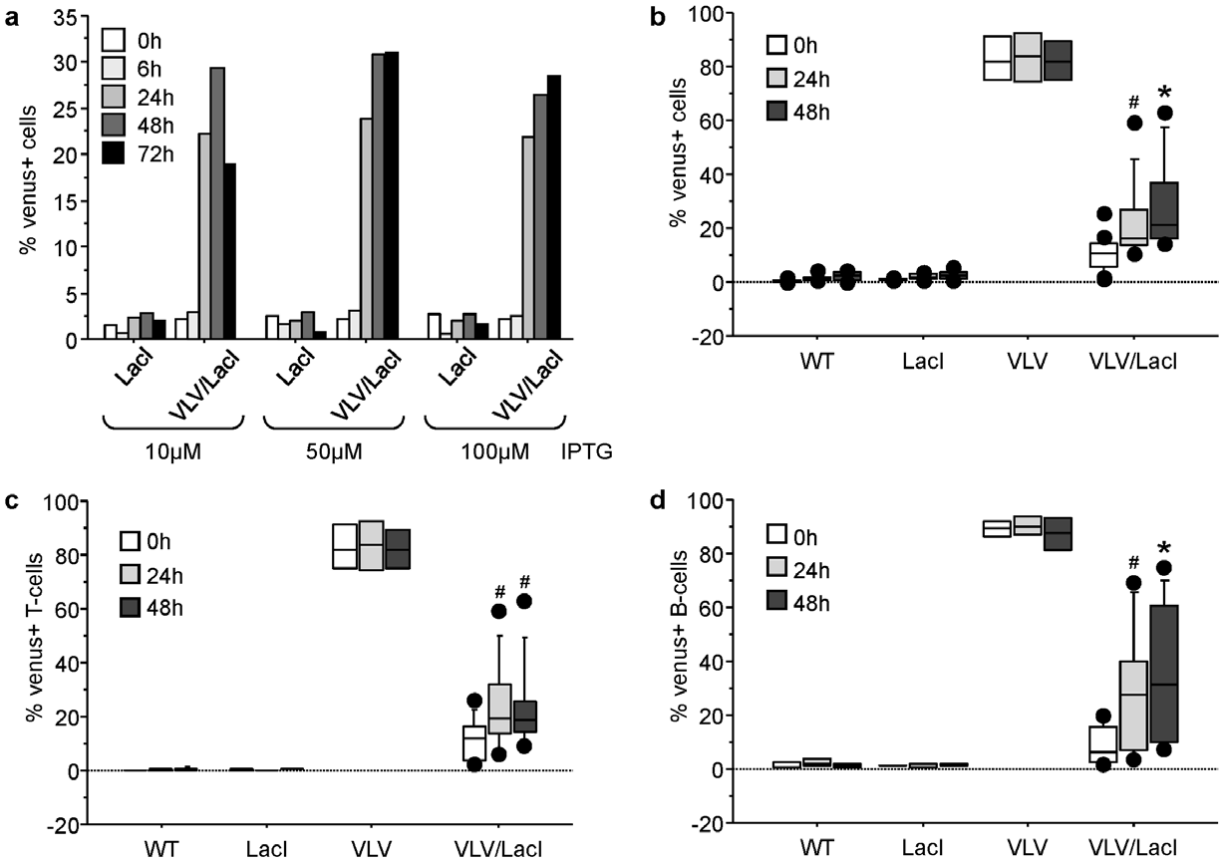
Transgene silencing and re-expression of transgene expression *in vitro*. Primary cells derived from peripheral blood of VLV/LacI double transgenic mice were put in culture and stimulated with graded doses of IPTG. FACS analysis was performed at the indicated time points to monitor levels of Venus^+^ cells (**a**). Peripheral blood lymphocytes from mice of the indicated genotypes were stimulated with IL-2+PMA+ionomycin to drive T-cell activation or IL-2+IL-4+IL-5+LPS to drive B-cell proliferation. Proliferating cells were treated with 50 µM IPTG on day 3 after mitogen stimulation and levels of Venus^+^ cells were quantified by FACS analysis on all cells (**b**), as well as T-cells (**c**) or B-cells (**d**). Results from four independent experiments and 4-9 animals/genotype (wt = 5, LacI n = 5, VLV n = 4, VLV/LacI n = 9) are represented as box plots. Box length equals interquartile range. Circles represent minimal and maximal values; *p<0.001; #p<0.05. F2 generation offspring of VLV founder A3 were crossed with LacI mice. Single-, double-, and non-transgenic littermates were used for analysis.

After having characterized Venus expression in the single transgenic mice, we started to cross VLV mice with mice transgenic for *lacI*. In these animals the Lac repressor protein is ubiquitously expressed from the human β-actin promoter, with high levels of repressor protein detected in the spleen [10]. First we started to analyze if Venus expression was shut down effectively in the peripheral blood of double-transgenic mice identified by PCR genotyping, using flow cytometric analysis and whether it was re-inducible in culture. Venus expression in the peripheral blood dropped to ∼5% in double-transgenic animals, indicating effective shut down of transgene expression. We were also able to re-induce Venus expression upon IPTG treatment in a significant portion of the cells (p<0.05), monitored up to 72 h and reaching plateau after 48 h with up to 30% of the cells re-expressing Venus (Fig. 5a). The dose-response behaviour of the cells suggested that the system was already maximally induced with 10 µM of IPTG, but Venus expression levels appeared more stable at later time-points when ≥50 µM IPTG were used, the concentration we chose for further experiments. When monitoring the percentage of mitogen-induced Venus^+^ T or B cells blasts derived from peripheral blood over time by gating on TCRβ^+^ T or CD19^+^ B cells, respectively, no significant difference was observed between the cell types (p = 0.23), but we were unable to reach the percentage of Venus^+^ cells observed in the peripheral blood from single transgenic VLV mice (Figs. 5b–d). Extended culture of cells was not increasing the percentage of Venus^+^ cells, probably due to the high rate of cell death occurring in the T and B cell blasts over extended times *ex vivo* which ultimately associated with loss of Venus expression (not shown).

**Figure 6:**
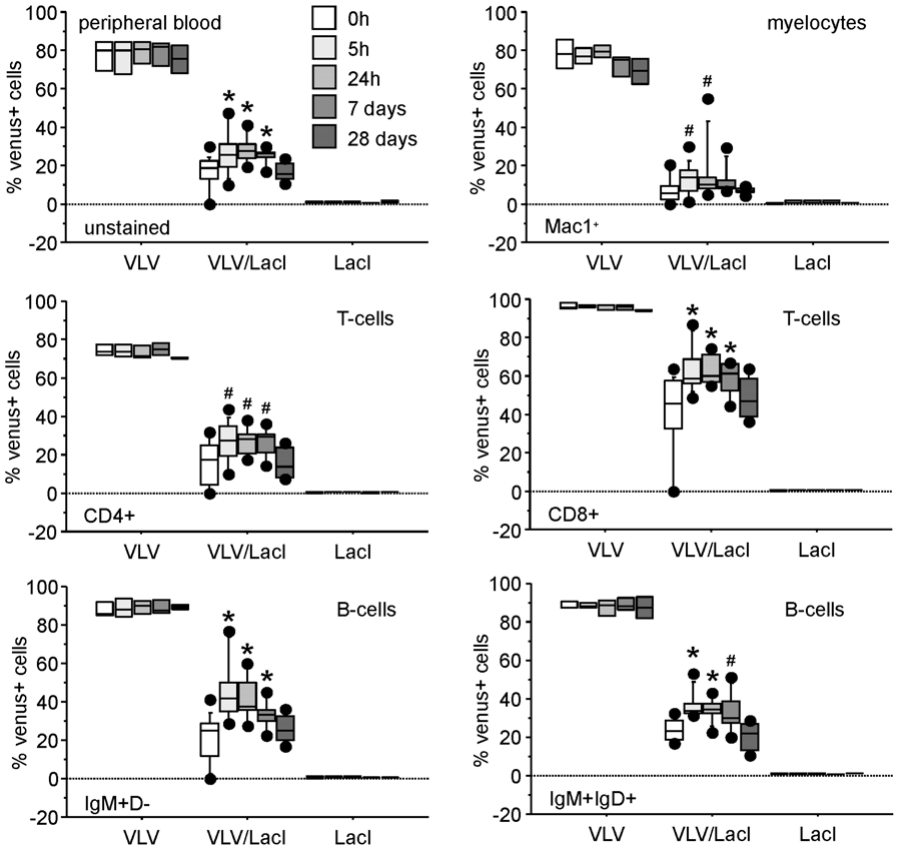
Transgene re-expression *in vivo*. Mice of the indicated genotypes were treated *i.p.* with 1 mg IPTG and levels of Venus^+^ cells in T-, B- and myeloid subsets in the peripheral blood were quantified by FACS analysis following the indicated times. Results of three independent experiments and 3 (VLV and LacI) or 6 (VLV/LacI) animals/genotype are represented as box plots. Box length equals interquartile range. Circles represent minimal and maximal values; *p<0.001; #p<0.05. F2 generation offspring of VLV founder A3 were crossed with LacI mice. Single-, double-, and non-transgenic littermates were used for analysis.

Next, we aimed to verify whether the system was inducible *in vivo*. First, we started to treat the animals with 10 mM IPTG in the drinking water, as suggested by previous results using a regulated version of the *tyrosinase* gene [10]. However, after monitoring Venus expression after 1 and 3 weeks of IPTG-treatment, respectively, we were unable to detect a significant portion of treatment-induced Venus^+^ cells in the peripheral blood of these animals. In line with our organ analysis, CD8^+^ T cells that showed highest levels of transgene expression (Fig. 4), showed best induction in a subset of cells (up to 10%), while induction in other T and B cell subset was detected in less than 5% of the cells (data not shown). So we decided to treat the mice with a single dose of IPTG *i.p.* In a pilot experiment, we treated 8 weeks old animals with graded doses of IPTG (0.1, 1 or 10 mg) and monitored the percentage of Venus^+^ cells in peripheral blood over time. As in our *in vitro* experiments, no substantial differences were noticed between the three doses tested and 1 mg IPTG was chosen for further experiments (data not shown). We collected peripheral blood from the tail vein of treated animals in different intervals and stained cells with antibodies specific for different cell surface markers identifying myelocytes, T and B cells. Similar to our *in vitro* experiments we were able to detect a significant increase (p<0.05) in percentage of Venus^+^ cells in the peripheral blood of double-transgenic mice, with CD8^+^ T cells and IgM^+^D^−^ naïve B cells showing the best response while Mac-1^+^ myelocytes only responded poorly, if at all (Fig. 6). However, variation in gene silencing was high with CD8^+^ T cells showing the highest leakiness in double transgenic mice. In none of the cell types analyzed were we able to induce Venus expressing to the levels noted in single transgenic mice (Fig. 6).

## Discussion

We adopted the expression plasmid *VavP21/45*, containing the promoter of the *Vav* gene, to drive expression of a fluorescent reporter protein, Venus, under the control of the *lac* operator *in vivo*. We confirmed functionality and regulation of reporter expression after insertion of *lacO* binding sites by LacI after IPTG treatment in 293T human embryonic kidney cells (Fig. 1). In the presence of a co-transfected Lac repressor, the percentage of Venus^+^ cells was reduced but not completely shut down, consistent with previous results ectopically expressing a *lacO*-modified version of the human *huntingtin* promoter driving firefly luciferase [10], [15]. Initially, we ascribed the limited expression and silencing to the fact that a heterologous cell system was used where the *Vav*-gene is usually not active [6]. Also, the addition of IPTG to the cell culture media only partially restored original levels of Venus expression in 293T cells, which has been noted before when using this system in mammalian cells [16]. Incomplete shut-down of transgene expression might be related or due to the spacing and positioning of the lacO elements in close proximity and short distance within the pOPI3-CAT-derived SV40 intron, upstream of the multiple cloning site. It has been reported before, that two *lacO* sites positioned on either site of the transcription start site are sufficient to confer regulation to mammalian promoters by lacI and IPTG, while the third binding side is usually positioned further up or downstream and mainly serves to increase the local concentration of lacI [11]. However, since we did not want to interfere with the tissue-specificity of the *VavP* promoter-construct, we were prepared to accept reduced promoter regulation in our initial *in vivo* approach using Venus as a reporter.

When assessing transgene expression in peripheral blood of the founders, we found a high variation in the percentage of Venus^+^ cells (Fig. 2), a possible consequence of the microinjection technique used to generate transgenic animals, where copy number and insertion sites can vary and mosaicism is frequently observed due to transgene insertion at the two cell stage or later. However, consistent with the heritable nature of position effect variegation (PEV) [17], transgene expression was also observed to vary in F2 progeny of selected founders. Based on our organ analysis, this appeared to vary strongly between cell types and tissues (Fig. 4). Our results resemble those reported initially when using the bacterial *β-gal* gene as a reporter in the context of the *VavP* promoter that was expressed in a variegated manner and subjected to frequent silencing over time [18]. PEV is a well known phenomenon in transgenesis depending on chromatin structures near the site of insertion, most pronounced when transgene constructs are used that lack a defined locus control region [17], but also hard to distinguish in our case from gene silencing-induced mosaicism. Based on experiences using hCD4 or hBcl-2 as transgenes in the *VavP* vector showing uniform expression of the human transgenes tested [6], [7], we can only speculate that the non-mammalian *Venus* sequence, although codon optimized for the use in mammalian cells, may be susceptible to stochastic epigenetic silencing in a certain percentage of cells, as was observed previously when *VavP-lacZ* tg mice were compared to *VavP-hCD4* tg mice [6], [7]. Alternatively, the pOPI3-CAT derived SV40-derived intronic sequence containing the lacO sites may be subjected to epigenetic silencing, as noted before when bacterial or viral sequences were introduced into mammalian genome [19]. However, the latter seems unlikely, since we noted similar patterns of transgene expression in the VV strain, lacking this DNA element (Fig. 5). Since we were forced to use different restriction enzymes to linearize the modified VLV plasmid, it remains also possible that the residual bacterial DNA sequence of about 250 base pairs that we could no longer remove (Fig. 1a) may render the transgene more susceptible to silencing. Alternatively, high-copy number insertion of transgenes reportedly favours variegation of expression due to repeat-induced gene silencing [17] which may in part explain why VV mice, that do have a higher copy number insertion than VLV mice based on qPCR analysis (not shown), showed stronger variegation (Fig. 4). Alternatively, high-level expression of Venus may not be well tolerated in haematopoietic cells causing silencing of transgene expression over time or counter selection in stem cells. High-level GFP expression was reported to induce apoptosis in cultured cells and cardiomyopathy was observed in FVB mice with high-level expression of GFP when driven from the cardiac alpha-myosin heavy chain promoter [14]. However, our *in vitro* analysis did not show increased cell death in Venus expressing lymphocytes (Fig. 2) and counter selection due to toxicity in leukocytes can be largely excluded since we did not see a significant reduction of the percentage of Venus^+^ cells in peripheral blood over time (not shown).

When we crossed our single transgenic VLV mice with the *LacI* repressor mice we noticed that reporter transgene expression was actually quite effectively silenced in the peripheral blood where only about 5% of cells scored as Venus^+^ by highly sensitive flow cytometry. In addition, emission of Venus fluorescence is significantly stronger as the one emitted by GFP [12], which initially suggested that silencing is very effective and well in the range or even better as when the Bujard system was used *in vivo* [20]. This background expression of Venus did not differ significantly between cell types of the peripheral blood (Fig. 4) making broad differences in *lacI* expression across the different haematopoietic cell types analyzed unlikely. However, when testing for the ability to re-induce transgene expression *in vitro*, we noted that we were unable to reach more than 30% Venus^+^ T or B cell blasts (Fig. 5). While this may have been due to high rates of cell death that correlates with loss of Venus expression in primary lymphocytes upon extended *in vitro* culture, application of IPTG in the drinking water also failed to drive transgene re-expression in peripheral blood (not shown). This may be due to the reported short half-life and rapid clearance of IPTG from the peripheral blood stream by excretion via filtration in the kidneys [21] but should not be an issue *in vitro*. Similarly, when injecting mice with IPTG *i.p.*, we also noted a very limited response, combined with considerable leakiness in different cell types that again differed between individual animals (Fig. 6). Notably, either repression was good, i.e. less than 10% of the cells were Venus^+^ then re-induction was also poor (20–30%), or the system was leaky (>20% Venus^+^ cells) allowing high levels of re-induction (up to 80%, e.g. in CD8^+^ T-cells). This opens the possibility that the inefficient shut down and leakiness observed may be related or due to inefficient expression of *lacI* by the *β-actin* promoter in different haematopoietic cell types. In previous studies characterizing *LacI* tg mice the levels of LacI protein expression was only assessed in tissue lysates but not at the cellular level [10], [15], leaving the possibility that its expression may actually be also not uniform across cell types within tissues. Alternatively, the *Vav*-gene promoter that shows differences in activity in between leukocyte subsets and is highly susceptible to epigenetic regulation [18] may either be too weak to overcome the repressive effect of LacI in all cells of a given lymphocyte subset before clearance of the inducer, or, become more easily subjected to heterochromatinization e.g., due to lacI-mediated inefficient transgene expression, favouring such events in some type of negative feed back loop. However, this needs to be formally demonstrated.

Our findings using the *lacO/lacI* system in haematopoietic cells differ from the successful use of this system driving tyrosinase expression in pigment-producing melanocytes, suggesting that these cells are able to accumulate more inducer and maybe retain higher level/constant expression of LacI. Furthermore, re-expression of the transgene in a subset of hair follicle stem cells or derived melanocytes may actually be sufficient to restore pigmentation [10].

Although, the *lacO/lacI* system in the current version tested shows only limited suitability for regulated transgenesis in haematopoietic cells, after optimization, e.g., of lacO element number/positioning and/or reduction of transgene copy number, it may still be useful for certain applications. Variegated or mosaic expression of Venus may even be exploited to target expression of pro-apoptotic genes, recombinases or transforming oncogenes only to a subset of cells, e.g., by inserting an IRES-element in the 3′UTR of *Venus*, followed by the cDNA of interest. Alternatively, the same trick can be exploited to introduce an RNA interference based gene-silencing cassette, eliminating expression of a target gene in a subset of cells.

## Materials and Methods

### Generation of transgenic mice & vector manipulation

Animal experiments were in accordance with Austrian law and approved by the Austrian Ministry for Science and Education (BMBWK-66011/0136-BrGT/2006). A 484 bp SV40-derived intronic sequence fragment derived from the pOPI3CAT plasmid (Stratagene), containing 3 symmetric *lacO* sites (http://www.genomics.agilent.com/files/Vectors/Sequences/opi3cat_s.txt) was excised by BglII digest and subcloned after fill-in and NotI digest into partially NaeI, EagI digested *VavP21/45* transgenic vector. The transgenic lines *VavLacOVenus* and *VavVenus* were generated by microinjection of a 9.2 kb AseI-PvuI fragment gel purified from the corresponding plasmids. The DNA was microinjected at approximately 2–3 ng/µl into the pronuclei of fertilized oocytes from FVB mice (Charles River Laboratories) at the one-cell stage following standard procedures. Founders containing >80% of *Venus^+^* cells in the peripheral blood were chosen to establish transgenic lines. Due to variegated transgene expression, the percentage of Venus^+^ cells was determined from peripheral blood and only mice with >80% of Venus^+^ cells in F2 were used for further breeding with non-transgenic mice to maintain a hemizygous state. Double-transgenic mice were generated by intercrossing *VLV* mice with FVB β-actin *LacI* transgenic mice [10].

### DNA extraction and PCR typing

DNA was extracted from tail tips digested overnight at 55°C in 500 µL of 50 mMTris (pH 8), 200 mM NaCl, 0.5% SDS, 5 mM EDTA and 2 µg/ml Proteinase K. Undigested material was sedimented by centrifugation and 350 µL was transferred into a clean 1.5 ml tube. DNA was extracted by isopropanol precipitation (1∶1; v/v) followed by centrifugation in a microfuge (2 min, 13.000 rpm, 4°C). The pellet was washed once in 1 ml 70% ethanol. After another centrifugation step, the pellet was air-dried and resuspended in 100 µL TE at 55°C for about half an hour. 1 µl was used per PCR reaction.

Littermates from heterozygous *VavLacOVenus* or *VavVenus* mice were genotyped on tail DNA by PCR using primer pairs specific for SV40 polyA sequence and the Venus coding sequence, respectively. The following cycle conditions were used: 5 min at 94°C; 40 s at 94°C, 30 s at 60°C, and 3 min at 72°C (35 cycles); and 10 min at 72°C. *VavLacOVenus* allele primers: forward, 5_-CCTAGGTTGTGGAATTGTGAG-3, and reverse 5_- CCAGGGCACGGGCAGCTT-3, (330 bp product); VavVenus allele primers: forward, 5_-GCCTGCAGTGCTTCGCCCGC-3, and reverse 5_-GCTTGTCGGCGGTGATATAGACG-3, (250 bp product).

Littermates from heterozygous *VLV/LacI* double transgenic mice were genotyped by PCR using primer pairs specific for SV40/Venus sequence (as described above) and *lacI* respectively. The following cycle conditions were used for *lacI* PCR: 5 min at 95°C; 30 s at 95°C, 15 s at 63°C, and 1 min at 72°C (30 cycles); and 10 min at 72°C. *lacI* allele primers: forward, 5_-GCACTCCAGTCACCTTCTCTTTCA-3, and reverse 5_-TGGGAGCCTCTG TGGTGGTGTCAA-3, (450 bp product).

### IPTG treatment *in vivo* and peripheral blood sampling

IPTG (SIGMA-Aldrich) was dissolved in sterile water to a final concentration of 5 mg/ml. Animals were injected *i.p.* with 1 mg IPTG in 200 µl of a freshly prepared solution or IPTG was provided in drinking water 10 mM/1%glucose in light-protected bottles, changed in 2–3 day intervals. Mice were anesthetized by Isofluorane inhalation (ABBOTT Laboratories) and then peripheral blood was collected from a lateral tail vein or the retrobulbar vein plexus. Heparin (Ebewe Pharma) was added to the 1.5 ml collection tube (10 µl) to avoid blood coagulation.

### Generation of T- or B-cells blasts and IPTG treatment *in vitro*

Samples from peripheral blood were subjected to red blood cell lysis for 5 minutes at 37°C followed by repeated washing in 1 ml PBS and centrifugation (4°C, 5 minutes, 1500 rpm). Cell pellets were resuspended in RPMI 1640 medium, supplemented with 250 mM L-glutamine, 50 µM 2-mercaptoethanol, penicillin/streptomycin (1 U/ml), 10% FCS, non-essential amino acids (Invitrogen) and 1 mM pyruvate (Invitrogen). For the generation of T-cell blasts, medium was complimented with 100 U/ml of mIL-2 (Peprotech) and 2 µg/ml Concanavalin A (Sigma-Aldrich). For the generation of B-cell blasts, medium was complemented with 100 U/ml of mIL-2, 10 µg/ml mIL-4, 10 µg/ml mIL-5 (all Peprotech) and 20 µg/ml LPS (Sigma-Aldrich). Cells were cultured for 3 days and allowed to form blasts prior stimulation with 50 µM IPTG. Aliquots of the cultured cells were recovered, centrifuged (5 min, 1500 rpm, RT) and washed once in excess PBS prior antibody staining and flow cytometric analysis. Cell pellets were resuspended in 200 µl of PBS containing 2% rat serum plus either anti-TCRβ-PE or anti-CD19-PE (Biolegend), diluted 1∶200 and incubated for at least 30 min on ice in the dark. Flow cytometric analysis was performed to monitor induction of Venus expression in T or B cell blasts over time.

### Cell culture and transient transfection

Cells were cultured at 37°C in a humidified atmosphere containing 5% CO_2_. 293 T human embryonic kidney cells (ATCC, CRL-11269) were cultured in the DME medium supplemented with 10% FCS (PAA), 250 µM L-glutamine (Invitrogen) and penicillin/streptomycin (1 U/ml, Sigma-Aldrich). Primary thymocytes, lymph node and spleen cells were cultured in RPMI 1640 medium, supplemented with 10% FCS (PAA), 250 mM L-glutamine (Invitrogen), 50 µM 2-mercaptoethanol, penicillin/streptomycin (1 U/ml, Sigma-Aldrich), non-essential amino acids (Invitrogen) and 1 mM pyruvate (Invitrogen).

293T human embryonic kidney cells were transiently transfected in 6-well plates using 3ìl of Lipofectamine 2000 reagent (Invitrogen) and a total of 1ìg of plasmid DNA. Cells were seeded at a density of 1×10^5^/well the night before. During transfection cell were incubated in 1 ml Optimem (Gibco) and 250 µl of Lipofectamin (Invitrogen) mix was added for 6–16 h before replacing Optimem with regular culture medium after a single wash with PBS. After culture for 24 h, IPTG (tested in a range between 0.1 µM–1 mM) was added for additional 24 h. After 48 h cells were collected by trypsinization, along with their supernatant and centrifuged for 5 minutes, at 4°C, 1500 rpm. Pellets were then resuspended in PBS and flow cytometric analysis was performed to determine percentage of Venus positive cells using a FACS_Calibur instrument (Becton Dickinson).

### Immunoblotting

Mouse tissues were flushed in PBS, snap frozen on dry ice and subsequently lysed in RIPA buffer containing protease inhibitors (Roche) in a tissue grinder (MP Biochmedicals) set at 6 m/sec for 40 seconds using FAST-Prep lysis matrix D containing silica spheres (MP Biochmedicals) in a 2∶1 v/w ratio. Suspensions were kept on ice for 60 min, insoluble material was spun down in a microfuge (3 min, 13.000 rpm, 4°C) and the supernatant transferred into fresh tubes. Aliquots of lysates were used for protein quantification by Bradford (Biorad), stored at −80°C. 50 µg of total protein extracts were separated denatured and boiled in 5× Laemmli sample buffer prior SDS-PAGE on 12% Tris-Glycine gels and electroblotted (X-cell, invitrogen) onto nitrocellulose membranes (Amersham). Membranes were probed with rabbit anti-GFP antiserum and mouse anti-GAPDH mAb (Cell Signaling, 14C10). Horseradish peroxidase (HRP)-conjugated goat anti-rabbit or rabbit anti-mouse antibodies (DAKO) served as secondary reagents and the enhanced chemiluminiscence (ECL; Amersham) system was used for detection.

### Cell death assays

Primary lymph node cells, thymocytes and splenocytes derived from transgenic mice isolated after sacrifice were kept in culture as indicated above and percentage of viable cells was determined by staining with 2 µg/mL propidium iodide plus AnnexinV-APC (Biolegend) in annexin binding buffer (0.1 M HEPES/NaOH, pH7.4; 140 mM NaCL; 25 mM CaCl2) and analyzing the samples using a FACS_Calibur instrument (Becton Dickinson).

### Flow cytometric analysis

Single-cell suspensions from peripheral blood, thymus, spleen, inguinal lymph nodes and bone marrow were surface stained with monoclonal antibodies conjugated with R-PE, APC or biotin in 300 µl of PBS containing 2% rat serum plus antibody diluted 1∶200 and incubated for at least 30 min on ice in the dark. If necessary, cells were subjected to a second incubation step after a washing step using streptavidin-RPE or streptavidin-PE-Cy7 (both from DAKO) diluted 1∶200 in PBS. The monoclonal antibodies used and their specificities are as follows: GK1.5, anti-CD4; 3C7, anti-CD25; RB6-8C5, anti-Gr-1 (all Biolegend); MI/70, anti-MAC-1; 11/26C, anti-IgD; R2/60, anti-CD43; MB19-1, anti-CD19; II/41, anti-IgM 53-6.7, anti-CD8; (all eBioscience). Biotinylated antibodies were detected using streptavidin-RPE (DAKO) or streptavidin-PE-Cy7 (Becton Dickinson). Flow cytometric analysis was performed using a FACS_Calibur cell analyzer (BD Biosciences) and 100.000-200.000 events were acquired for analysis.

### Statistical Analysis

Statistical analysis was performed using the unpaired *Student t test* and a Stat-view 4.1 software program. P-values of <0.05 were considered to indicate statistically significant differences.
